# Which features of patients are morally relevant in ventilator triage? A survey of the UK public

**DOI:** 10.1186/s12910-022-00773-0

**Published:** 2022-03-25

**Authors:** Lok Chan, Jana Schaich Borg, Vincent Conitzer, Dominic Wilkinson, Julian Savulescu, Hazem Zohny, Walter Sinnott-Armstrong

**Affiliations:** 1 Social Science Research Institute, Duke University, Durham, USA; 2 Department of Computer Science, Duke University, Durham, USA; 3 Department of Economics, Duke University, Durham, USA; 4 Department of Philosophy, Duke University, Durham, USA; 5 Department of Computer Science, Institute for Ethics in AI, University of Oxford, Oxford, UK; 6 Department of Philosophy, Institute for Ethics in AI, University of Oxford, Oxford, UK; 7 The Oxford Uehiro Centre for Practical Ethics, Oxford, UK; 8 Murdoch Children’s Research Institute, Melbourne, Australia; 9 John Radcliffe Hospital, Oxford, UK; 10 Kenan Institute for Ethics, Duke University, Durham, USA

**Keywords:** COVID-19, Medical ethics, Triage, Rationing, Scarce resources

## Abstract

**Background:**

In the early stages of the COVID-19 pandemic, many health systems, including those in the UK, developed triage guidelines to manage severe shortages of ventilators. At present, there is an insufficient understanding of how the public views these guidelines, and little evidence on which features of a patient the public believe should and should not be considered in ventilator triage.

**Methods:**

Two surveys were conducted with representative UK samples. In the first survey, 525 participants were asked in an open-ended format to provide features they thought should and should not be considered in allocating ventilators for COVID-19 patients when not enough ventilators are available. In the second survey, 505 participants were presented with 30 features identified from the first study, and were asked if these features should count in favour of a patient with the feature getting a ventilator, count against the patient, or neither. Statistical tests were conducted to determine if a feature was generally considered by participants as morally relevant and whether its mean was non-neutral.

**Results:**

In Survey 1, the features of a patient most frequently cited as being morally relevant to determining who would receive access to ventilators were age, general health, prospect of recovery, having dependents, and the severity of COVID symptoms. The features most frequently cited as being morally irrelevant to determining who would receive access to ventilators are race, gender, economic status, religion, social status, age, sexual orientation, and career. In Survey 2, the top three features that participants thought should count in favour of receiving a ventilator were pregnancy, having a chance of dying soon, and having waited for a long time. The top three features that participants thought should count against a patient receiving a ventilator were having committed violent crimes in the past, having unnecessarily engaged in activities with a high risk of COVID-19 infection, and a low chance of survival.

**Conclusions:**

The public generally agreed with existing UK guidelines that allocate ventilators according to medical benefits and that aim to avoid discrimination based on demographic features such as race and gender. However, many participants expressed potentially non-utilitarian concerns, such as inclining to deprioritise ventilator allocation to those who had a criminal history or who contracted the virus by needlessly engaging in high-risk activities.

**Supplementary Information:**

The online version contains supplementary material available at 10.1186/s12910-022-00773-0.

## Introduction

Patients infected by SARS-CoV2 often develop hypoxic respiratory failure and may die if they are not put on a mechanical ventilator in an intensive care unit (ICU) bed [1]. The large number of patients simultaneously needing breathing support during the coronavirus pandemic has put immense pressure on healthcare systems worldwide.

In the early stages of the pandemic, bioethicists, healthcare experts, and practitioners across the world proposed various guidelines for how healthcare resources, including ventilators, should be distributed when their supply is severely limited. An international study found that virtually all triage guidelines appeal to the utilitarian consideration of maximizing benefits [2]. For example, the guidelines provided by British Medical Association, published in April 2020, state that the guiding principle for triage (when necessary) is “the greatest medical benefit to the greatest number of people” [7]. Similarly, the International Society of Critical Care Medicine (SCCM) also recommends that the allocation of scarce medical resources should be “based on the premise of the greatest good for the greatest number” [8]. Likewise, a study in the U.S. found that hospitals with a triage policy primarily use medical benefit as one of the main triage criteria [3].

There has been less consensus about what to do when giving a ventilator to one patient will not yield greater medical benefits than giving it to another. In one approach, the Australian Government Department of Health specifies that such triage decisions should be made so that vulnerable groups, people with dependents to care for, and frontline medical workers are prioritized over others [6]. In another approach, an expert panel of the Task Force for Mass Critical Care and the American College of Chest Physicians suggests that children or pregnant women should receive priority on the ground that saving their lives will likely lead to greater life-years saved overall [5].

Here, we assess which features of COVID-19 patients the public in the United Kingdom think should be used in ventilator triage. We contrast their priorities with the guideline provided by the British Medical Association to assess whether published professional guidance coincides with the values of the wider community. According to the guideline, if triage becomes necessary due to the severe constraint of medical resources, the following features of a patient should be considered: probability of survival, prospect of recovery, co-morbidity, frailty, patient wish, and being an essential worker [7]. The authors of the guideline acknowledge that this list is not directly medical. The proposal that essential workers ought to be prioritized, for example, cannot be grounded purely in terms of medical utility for the treated patient, and instead draws on additional ethical values such as the maintenance of the social good.

Another issue in triage decisions is whether or not there are features that it would be wrong to consider even when medical resources are limited. The British Medical Association cites equality and fairness as a guiding principle: “everyone matters equally”, implying that a patient’s gender, economic status, race, etc., should not be considered when allocating medical resources. The guideline also explicitly states that the decision to provide or withhold resources should not be based on age or disability, as they do not determine the degree or probability of medical utility from receiving the resource, e.g., a younger patient could have lower chance of survival than an older patient due to comorbidities. The guideline also states that unrelated health conditions or impairments, such as learning disabilities, should not be considered.

To gauge the public’s views on triage, then, we must consider both which features they think should be considered and which they think should not. Previous studies on views of lay people on medical resources allocation have been carried out (for example, [21]) and scarcity of medical resources existed even before the current pandemic. Nevertheless, people’s exposure to resource scarcity and its cause due to this pandemic provided a rare opportunity to assess these public opinions at a time of heighted awareness of this problem. We do not claim that we should directly draw normative conclusions from these public opinions, but the views of the public are relevant to policy makers who may wish to be responsive to the values of the community.

Our research was conducted in two stages. In the first stage, survey participants were asked in an open-ended survey to provide a set of features that they thought should be considered and a set of features that they thought should not be considered in ventilator triage (Study 1). In the second stage, a separate group of participants were asked to indicate how strongly they thought features identified in Study 1 should count in favour of or against a patient in need of a ventilator (Study 2).

We found that participants generally agreed with the British Medical Association’s guideline to focus on directly medical features and avoid discrimination. However, a considerable portion of the participants were also in favour of going beyond general utilitarian concerns and beyond the British Medical Association’s current guideline for ventilator triage by, for example, deprioritizing ventilator access to patients that needlessly engage in high-risk activities or prioritizing patients with dependents.

## Study 1

### Participants

Participants were recruited through Qualtrics between 10 and 23 June 2020. Participants were sampled to be representative of UK population for age (18–24: 21%; 25–34: 19%; 35–44: 18%; 45–54:20%; 55–64:17%; 65+:14) and gender (51% female). After excluding those who failed either a simple attention check (in which participants were explicitly asked to choose an option out of 5) or sampling criteria, the final sample included 525 UK residents. Participants were paid between £0.78 and £2.18, in accordance with the duration of the survey and the minimum wage.

### Design

At the beginning of the survey, participants read an introductory paragraph that described how some COVID-19 patients will not survive without treatment on a ventilator and there might not be enough ventilators for all patients who need them, in which case a doctor will have to decide which patients will get to use the available ventilators.

Participants were then asked to respond to two questions. First, which features of patients should be considered when deciding who should get the ventilator? It was emphasized to participants that they should answer from “a moral point of view” so that a feature should be considered when it would be morally wrong not to consider the feature in the decision-making process. Second, participants were asked which features would be morally wrong to consider when deciding who should get the ventilator in the same triage situation. No prompting was given about which features to include, though an example was given that some might think that patient age was either a feature that should be included or should not be included in decisions.

For each question, participants were asked to type five features in an open-ended format. After entering the features, they were then instructed to explain in textboxes why they think the feature in question should (for question 1) or should not (for question 2) be morally relevant. The full text of the survey is included in the Additional file 1.

### Analysis

To transform participants’ textual responses from Study 1 into quantifiable variables, an external coder was recruited to sort the textual responses into feature categories. For instance, some participants responded that age should matter, while some responded that younger people should get priority. These entries all counted towards the feature “age”. To represent the prevalence of a feature category in the participants’ responses, we then counted and ranked the number of participants who mentioned this feature. We did not simply count how many times a feature was mentioned in total, as certain features were often mentioned repeatedly by a participant. Instead, each participant’s response was transformed into a set of dichotomous variables where each member corresponds to a feature, and its value is 1 when the said feature was mentioned by the participant and 0 otherwise. For example, suppose a participant suggested in two different entries that being a child and being elderly were morally relevant. Both entries would be encoded as “age”. In the transformed dataset, the participant under consideration would receive “1” under the age column.

### Results for study 1

**Fig. 1:**
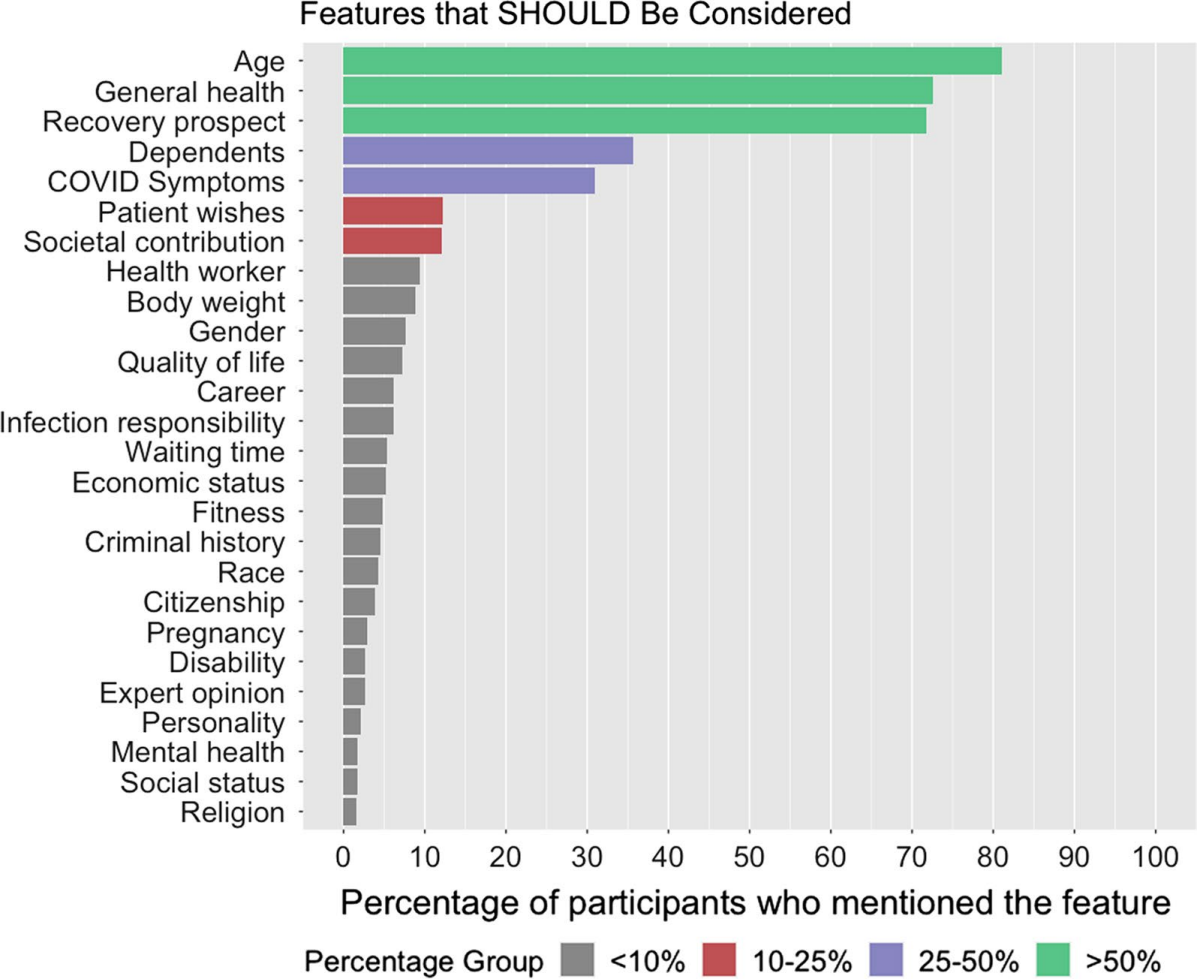
Features that participants believe should be considered in ventilator allocation decisions, organized by the percentage of participants who mentioned the feature (features mentioned by < 1% of participants not listed)

Table 1 contains the breakdown of features suggested by our participants that should and should not be considered, including the percentage of participants who mentioned this feature and the average age and gender distribution of these participants. To organize our description of results and accompanying data visualizations, we divided the features into 4 groups based on the percentage of participants endorsing the relevance of a factor: more than 50%, 25–50%, 10–25% and less than 10% (Fig. 1). Three features were endorsed by more than half of the respondents as features that should be considered in ventilator allocation decisions: age, general health, and prospect of recovery (supported by 81%, 73% and 72% respectively). When asked to explain why age was a morally relevant feature, 64% of explanations pointed to medical considerations such as life expectancy, prospect of recovery, and quality of life. Only 7% of the explanations involved an explicit appeal to non-medical considerations, such as the patient’s past contribution to society. When asked why they suggested that the general health of the patient should matter, 40% said it was because of a better chance of recovery and 15% cited life expectancy as the rationale.

**Table 1:** Features suggested by our participants that should and should not be considered, organized by the percentage of participants who mentioned this feature, and the average age and gender distribution of these participants (features mentioned by < 1% of participants not listed)

Feature	Participants %	Age: mean	Female—%	Male—%
*Features that should be considered*				
Age	81	44	49	50
General health	73	45	52	48
Recovery prospect	72	46	53	47
Dependents	36	45	49	50
COVID Symptoms	31	45	53	47
Patient wishes	12	47	59	41
Societal contribution	12	48	35	65
Health worker	9	44	33	67
Body weight	9	45	43	57
Gender	8	49	28	72
Quality of life	7	46	66	34
Career	6	44	31	69
Infection responsibility	6	42	41	56
Waiting time	5	39	54	46
Economic status	5	38	48	52
Fitness	5	48	44	56
Criminal history	5	45	29	71
Race	4	49	32	68
Citizenship	4	51	35	65
Pregnancy	3	43	47	53
Disability	3	48	43	57
Expert opinion	3	46	14	86
Personality	2	53	45	55
Mental health	2	51	33	67
Social status	2	46	22	78
Religion	2	41	38	62
*Features that should not be considered*				
Race	56	46	51	49
Gender	45	45	52	48
Economic Status	41	45	50	49
Religion	24	46	49	51
Social Status	24	46	49	50
Age	19	50	58	42
Sexual orientation	15	43	49	49
Career	13	44	48	52
Citizenship	10	50	39	61
General health	8	46	55	45
Dependents	7	47	49	51
Personality	6	43	50	50
Location	6	48	48	52
Disability	6	45	47	53
Criminal history	5	44	46	54
Body weight	5	42	67	33
Appearance	3	39	44	56
Political views	3	51	33	67
Lifestyle	3	49	81	19
Mental health	2	50	58	42
Patient wishes	2	52	42	58
Health worker	2	39	30	70
Expert opinion	2	46	62	38
Waiting time	2	42	50	50

The second most-highly endorsed group of characteristics included whether the patients have dependents (36%) and the severity of the patients’ COVID symptoms (31%). The third most-highly endorsed group of characteristics included the patient’s own wishes (12%) and the patient’s past or potential contribution to society (12%). Nineteen other features were mentioned by 1–10% of the participants.

**Fig. 2:**
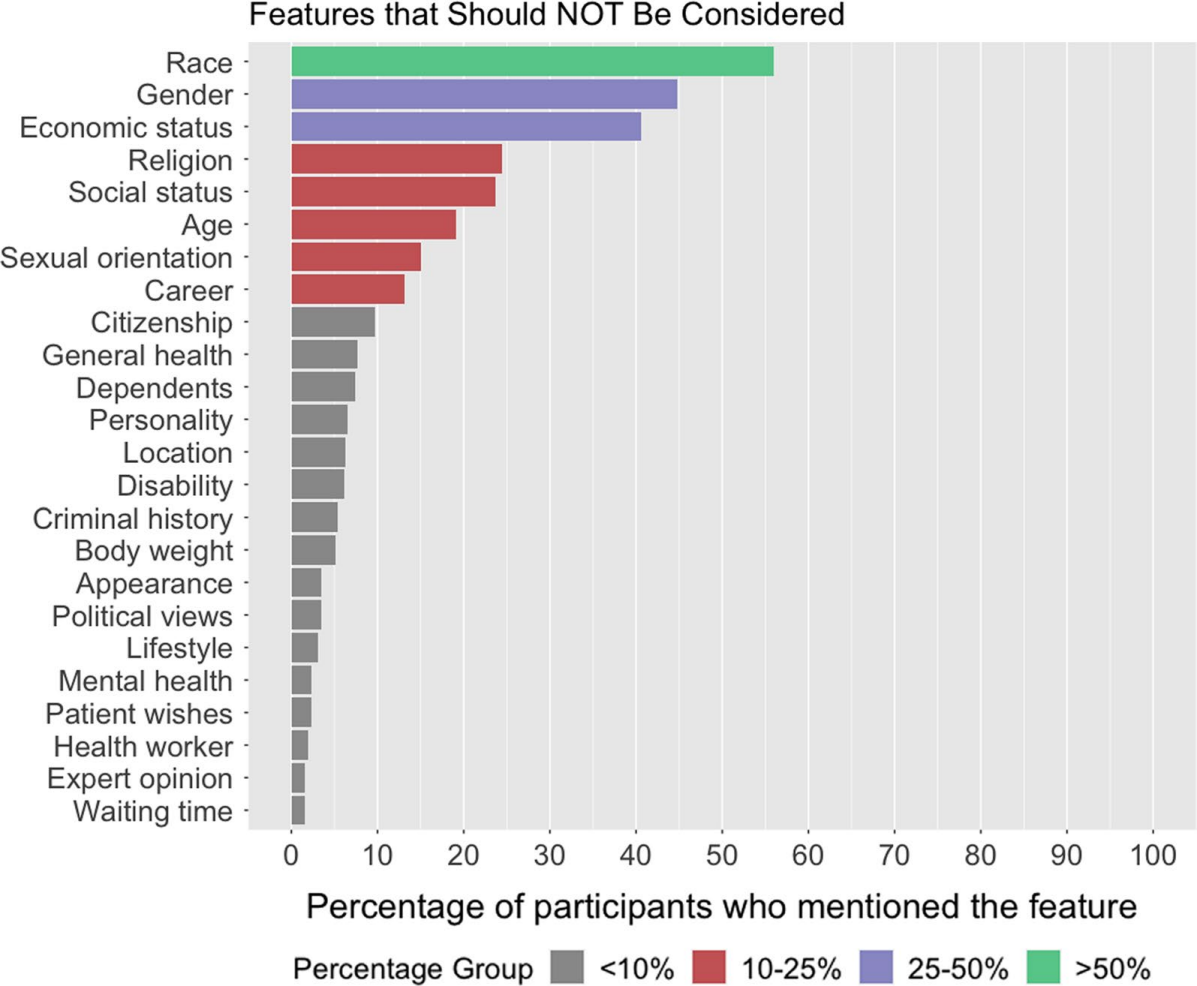
Features that participants believe should not be considered in ventilator allocation decisions, organized by the percentage of participants who mentioned the feature (features mentioned by < 1% of participants not listed)

Participants’ answers for features that should not be considered in ventilator allocation decisions were organized into the same 4 groups described previously for reporting and visualization (Fig. 2). Only one feature was identified by more than 50%: 56% of participants answered that race should be morally irrelevant. Next, 45% stated that gender should not be considered, and 41% suggested that the economic status of the patient (e.g., being rich/poor) should not be considered. Other features mentioned by more than 10% of respondents were religion (24.4%), social status (23.7%), age (19.1%), sexual orientation (15.1%), and career (13.2%). Those who said that career should not be considered did not mention whether or not they would allow essential workers as an exception.

Lastly, ten participants explicitly refused to provide any feature that they thought should be morally relevant. Five of these participants explained that the very idea of a picking a relevant feature was unfair or discriminatory. Three participants explained that their lack of answer was due to insufficient knowledge in the domain. Two participants did not explain their answers.

### Discussion for study 1

In this open-ended survey, features cited spontaneously by members of the UK public overlapped with those cited by professional guidance in the UK. Some of the top responses for morally relevant features, such as general health prospect of recovery and severity of symptoms, are in line with the guideline. In addition, the features cited by participants as not relevant, such as race, gender, economic status, and religion, are in line with principles of equality and fairness cited in the existing guideline.

There are two important exceptions: dependents and contribution to society, both of which are, at least on the face of it, not directly medical. Most participants who said having dependents is morally relevant did not explain their reasoning, but it is possible that their answer was motivated by utilitarian considerations: if a patient with dependents receives a ventilator, we might save not only the patient’s life, but also promote the well-being of those dependent on the patient. Nevertheless, as this study was not designed to specifically probe the underlying philosophical motivations of our participants, whose views could be consistent with non-utilitarian interpretations and could be revised when exposed to additional information.

Nevertheless, a small number of participants who provided societal contribution as an answer seemed to have in mind something more concordant with the guideline. First, 3 out of 30 of those participants explained that by “contribution” they meant medical professionals and 2 out of 30 indicated that societal contribution is determined by how essential the patient’s job was. These interpretations (though only 5 out of 30) line up with the guideline’s statement that essential workers should be prioritized for the sake of the social good. Second, participants could be referring to the patient’s potential to make societal contributions in the future, which has a strong utilitarian interpretation. Yet another interpretation is that participants meant that whether a patient had made contribution in the past should be morally relevant, which is the least utilitarian reading of this finding. Unfortunately, most participants who suggested that contribution to society should be considered did not explain their reasoning, so it is unclear which of these interpretations were intended by participants.

The prevalence of age in participants’ responses is potentially in direct contradiction with the guideline provided by the British Medical Association, which explicitly stated that age should not be considered in medical resource allocation. That said, when asked why they believed age was relevant, participants cited life expectancy, probability of recovery, and quality of life, potentially implying that they were using age as a proxy for features that are considered to be medically relevant in the guidelines.

While criminal history did not rank high in either category—only 24 (4.6%) participants suggested that it should be considered and 28 (5.34%) said it should not, this feature was noteworthy since criminal history is one of the few negative features mentioned by the participants. We also speculated that the participants who gave opposite answers might have had different kinds of crimes in mind. For instance, participants who said that criminal history should be considered might have thought that violent criminals should be deprioritized, and participants who said it should not be considered might have been motivated by the idea that people should not be punished for committed minor crimes.

It is important to note that Study 1 merely elicited a list of features without revealing the degree of importance participants assigned to the features. So, the fact that the largest proportion of participants thought age should be relevant should not be confused with the idea that they thought age is more important than others. Age was simply the feature that most readily came to mind for most participants, perhaps because age was an example in our instructions.

While only ten participants (2%) explicitly refused to provide any relevant features, more participants might have done the same, if they were explicitly encouraged to reflect on whether or not there should any morally relevant feature at all. Further, a previous study on decision making in the context of organ allocation suggests that people often opt out of deciding even if they previously indicated that a feature was relevant, when the difference in the variable is not large enough [22]. Thus, it is possible that participants who stated that certain features are relevant might nevertheless believe that in many cases these features should not make be decisive unless there’s a large difference between the two patients.

## Study 2

To build on the findings of Study 1, a second survey was conducted to determine the degrees of importance the UK public assigned to each feature in ventilator allocation, and to confirm the direction of perceived importance. The primary goal of this study was to identify features whose relevance to, and direction of favourability towards, ventilator allocation was agreed upon by a significant proportion of participants. Even though policy documents almost always strictly focused on positive features, such an assumption could not be made about the public, so we also had to explicitly probe whether people thought a feature should count in favour or against a patient. The secondary goal was to identify features whose relevance to ventilator allocation was agreed upon by a significant proportion of participants, but whose direction of favourability was not uniform such that participants were divided on whether the features should count in favour of or against a patient. In addition, in Study 2 we tried to answer some questions raised by the findings of Study 1. For example, there was a question in Study 1 whether or not participants meant specifically violent crimes when they said criminal record should matter. Because of this, in Study 2 we distinguished patients with a violent criminal record from those with a non-violent one, and, as we shall see, provide evidence that participants responded to these features differently. Lastly, we were also interested in features that participants would perceive as counting neither in favour nor against a patient, since these features should be consistent with the participant responses in Study 1 on features that should not be considered.

### Participants

505 participants, all UK residents, were recruited through Qualtrics (50% female; median age 46; 86% white) between 10 November and 3 December 2020. The sample collected aimed to be representative of the UK general population for age and gender (same proportions described in Study 1). Participants were paid between £0.78 and £4.50, in accordance with the duration of the survey and the minimum wage.

### Design

As in Survey 1, participants were given background information about the need for ventilators in COVID-19 patients and asked to imagine a scenario in which a doctor must decide which of the two patients would receive the only available ventilator. Then participants were given a list of 30 features extracted from Survey 1 in a randomized order (Table 2) and asked to indicate how important each feature ought to be for the doctor’s decision. Features were always presented with a specific contrast. For example, in Survey 1, many participants stated that the patient’s age morally should be considered. In Survey 2, this feature was turned into a contrast between ages: “Some patients are younger, and others are older. Please indicate how important this feature is: the patient under consideration is older than the other patient who needs a ventilator.” Full text of the survey is available in the Additional file 1. In response to each feature prompt, participants were given the following possible responses:

- +3: This feature should count strongly in favour of the patient getting the ventilator.
- +2: This feature should count moderately in favour of the patient getting the ventilator.
- +1: This feature should count slightly in favour of the patient getting the ventilator.
- 0: This feature should not count at all either in favour of or against the patient getting the ventilator.
−1: This feature should count slightly against the patient getting the ventilator.
−2: This feature should count moderately against the patient getting the ventilator.
−3: This feature should count strongly against the patient getting the ventilator.

**Table 2:** Feature labels, descriptions, response means, and p-values for the two t-tests

Feature labels	Description: the patient under consideration…	Mean	*p* value 1	*p* value 2
Dying soon	…will die very soon without a ventilator	2.02	< 0.0001	< 0.0001
Pregnant	…is pregnant	1.98	< 0.0001	< 0.0001
Waited for a long time	…waited for a long time for a ventilator	1.66	< 0.0001	< 0.0001
Extreme discomfort	…is under extreme discomfort due to severe COVID-19 symptoms	1.62	< 0.0001	< 0.0001
COVID health worker	… is a healthcare professional who contracted COVID-19 from working with COVID-19 patients	1.44	< 0.0001	< 0.0001
Longer life expectancy	…is expected to live a long time after successful treatment	1.30	< 0.0001	< 0.0001
Dependents	…has several dependents	1.06	< 0.0001	< 0.0001
Patient wishes	…expressed a preference to be put on a ventilator	1.00	< 0.0001	< 0.0001
Already on ventilator	…is already on the only available ventilator	0.90	< 0.0001	< 0.0001
Non-COVID health worker	…is a healthcare professional but does not work with COVID-19 patients	0.84	0.1195	< 0.0001
UK citizen	…is a UK citizen	0.83	0.0291	< 0.0001
Physically fit	…is physically fit	0.63	0.0827	< 0.0001
Future contribution	…is likely to make valuable contributions to society in the future	0.58	< 0.0001	< 0.0001
Age (older)	…is older	0.47	< 0.0001	< 0.0001
Past contribution	…has made valuable contributions to society in the past	0.46	< 0.0001	< 0.0001
Disabled	…is moderately or severely disabled	0.44	0.0028	< 0.0001
Frail	…is moderately or severely frail (defined as having age-related loss of physical and mental function that makes them dependent on others)	0.41	< 0.0001	< 0.0001
Minority race	…is in a minority race	0.40	< 0.0001	< 0.0001
Long ventilator time	…is expected to need to stay on the ventilator for a particularly long time	0.35	0.0001	< 0.0001
Mental health	…suffers from moderate or severe mental health problems	0.32	< 0.0001	< 0.0001
Female	…is female	0.28	< 0.0001	< 0.0001
Politician	…is a politician	0.13	< 0.0001	0.0055
Obese	…is obese	0.12	0.8940	0.0478
Celebrity	…is a famous celebrity	0.10	< 0.0001	0.0162
Low quality of life	…is expected to have a lower quality of life even after successful treatment	0.03	< 0.0001	0.5816
Non-violent crime	…a serious but non-violent crime in the past	− 0.03	< 0.0001	0.4489
Low chance of survival	…has a lower chance of surviving even if treated with a ventilator	− 0.21	< 0.0001	0.0047
Infection responsibility	…is responsible for catching COVID-19	− 0.33	0.0065	< 0.0001
Unnecessary risky activities	…contracted COVID-19 from needless risky activities	− 0.41	< 0.0001	< 0.0001
Violent crime	…committed a violent crime in the past	− 0.65	< 0.0001	< 0.0001

The feature descriptions in column 2 were presented to the participants. Labels were not shown to participants

A participant’s response is referred to as positive when the participant thought the feature should count slightly to strongly in favour of the patient (a response of +1, +2, or +3), negative when slightly to strongly against the patient (a response of −1, −2, or −3), and neutral when it should count neither in favour or against the patient (a response of 0). P-value 1 is for the test of relevance. P-value 2 is for the test of non-neutrality.

### Analysis

We wanted to statistically determine that, for each feature, (1) whether or not the feature was judged uniformly to be morally relevant by participants, and (2) whether there was a consensus on whether the feature should be favourable or unfavourable. To accomplish this, we carried out a two-step analysis. First, we created a variable to capture a participant’s perception of relevance for each feature, which was defined as 0 when a participant responded that a certain feature “should count neither in favour or against a patient”) and 1 otherwise (any non-zero response, either “in favour” or “against”). A two-sided one sample *t*-test was used to test against the null hypothesis that the mean of this variable was equal to 0.5. Second, another two-sided one sample t-test was conducted on the participants’ actual responses (from −3 to 3) to test the null hypothesis that the mean of each feature is 0, that is, whether or not the average opinion of our participants for a feature was neutral. After Bonferroni correction for 30 comparisons, we set our significance level at 0.05/30 = 0.0017. With 505 participants, we had 91% power to observe a small effect of *d* = 0.3.

### Results

**Fig. 3:**
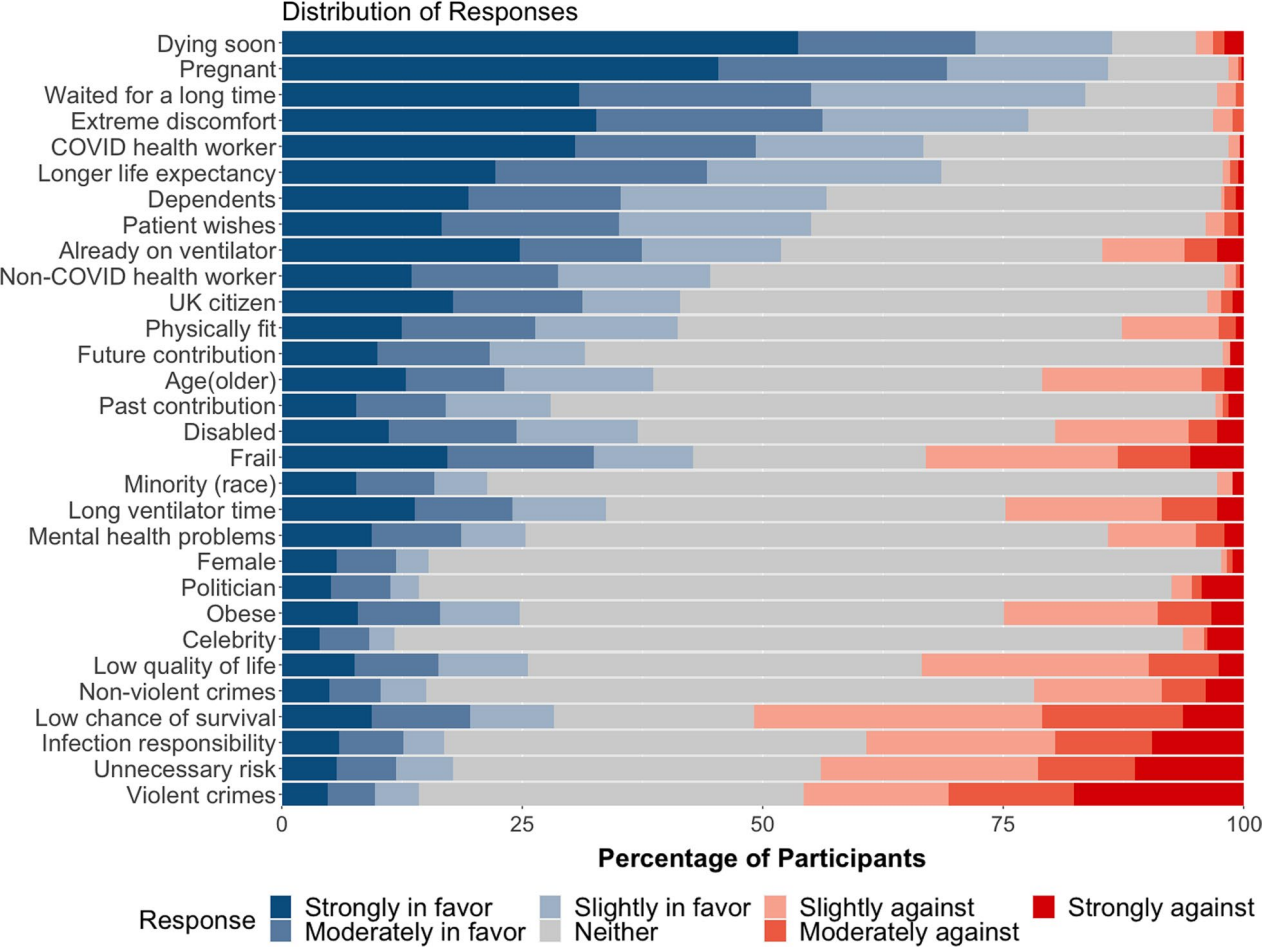
Distribution of response among participants, ordered by the average of all responses from highest to lowest

Figure 3 represents the distribution of all responses in percentages. Table 2 contains the description, mean, and raw counts of each feature, and the result of the statistical tests. The raw count for each response for each feature can be found on Additional file 1. The first t-test is a test of relevance: more specifically, it determines whether or not participants were divided in regard to this feature’s moral relevance, in which case the feature would fail to pass this test. In contrast, passing the first t-test suggests that there’s a statistically significant consensus among our participants whether or not this feature should matter in allocation. Six features failed the first t-test, indicating that participants were divided about the moral relevance of these six features: being responsible for getting infected, being obese, being disabled, being physically fit, being a UK citizen, and being a hospital worker who does not work with COVID patients. The rest of the features all passed the first t-test below 0.0017, the cutoff for significance after Bonferroni correction. The second t-test determined whether the mean response of a feature was statistically different than neutral. Six features failed the second t-test (indicating overall neutrality): obesity (the only feature to have failed both tests), being a famous celebrity, being a politician, having committed a non-violent crime, low quality of life, and low chance of survival, The rest of the features all passed the second t-test at significant level 0.0017, which indicates evidence that the mean for these features are not 0.

### Morally relevant and positive features

Nine features passed both t-tests with a positive majority, which is evidence that they were considered to be morally relevant and positive. These include: being pregnant (86% positive), dying soon (86%), waiting for a long time (84%), being under extreme discomfort (78%), being a COVID health worker (67%), having dependents (57%), wishing to be put on a ventilator (55%), and already on the only ventilator (52%). For these features, negative responses constituted less than 5% of total responses, except for patients who are already on the only ventilator, which has 15% negative responses.

### Morally relevant and negative features

Three features received primarily negative responses: low chance of survival, history of violent crimes, and needlessly engaging in risky behaviour. These three features passed the first t-test, indicating a consensus among participants regarding these features’ moral relevance. Interestingly, even though a patient with a low chance of survival received the highest percentage of negative responses (51%), it failed to achieve significance with Bonferroni correction (*p* value = 0.0047, which missed the cutoff of 0.0017). This was partly due to the relatively high number of positive responses, as 28% of participants thought that having a low chance of survival should in fact count in favour of the patient. In contrast, both history of violent crime and needlessly engaging in risky behaviour passed both t-tests, indicating that these negative opinions were statistically significant after Bonferroni correction.

### Divisive features

A feature that failed the first t-test can be considered as “divisive” in the sense that the participants were divided on whether or not the feature should be morally relevant. The dominant answer for all of these features was “should count neither in favour of nor against a patient”. They can be further organized in terms of the popularity of the non-neutral answers. First, some features were divided between favourable or neutral, such as being physically fit (41% vs 55%), being a UK citizen (41% vs 55%), and being a non-COVID hospital worker (45% vs 53%). Second, for some features, the non-neutral responses were more evenly divided, such as obesity (positive 25%, negative 25%, and 50% neutral). Third, infection responsibility is the only feature in this group that was divided along neutral and negative responses (44% vs 39%).

Quality of life may be also considered as a divisive feature in a unique way, as it passed the first t-test but failed the second. This suggests that even though the majority (59%) of participants provided non-neutral answers for these features, the responses were too divided for the balance of their opinions to point in one specific direction. While negative answers for this feature somewhat outnumbered the positives, it was not enough to be statistically significant.

### Morally irrelevant features

Eight features were perceived as morally irrelevant by our participants, in that while they passed the first t-test, suggesting that there was a consensus, the neutral answer dominated both positive and negative answer combined: being female, being a famous celebrity, being a politician, being a minority, past societal contributions, likely future contributions, history of non-violent crimes, and suffering from mental health problems. For each of these features, at least 60% of participants gave neutral answers.

### Study 2 discussion

As expected, our participants favoured prioritising direct medical utility to the patient. However, they also expressed a strong concern for features that do not directly lead to an increase in medical utilities by prioritising patients who waited for a long time and who have dependents. Three out of the four most negatively received features are not directly tied to medical utility. It is possible that participants were willing to deprioritise a patient based on their past behaviours (such as having committed a violent crime) or how they contracted the virus (such as engaging in a needlessly risky behaviour). It is also possible that the participants used a patient’s past risky behaviours as a predictor for a higher risk of future reinfection, in which case they might have been motivated by the concern that treating these patients would yield lower expected medical utility compared treating patients who are less likely to be reinfected.

We found a high degree of coherence between results from Study 1 and Study 2 regarding features that should not matter. Race and gender were the top two responses in Study 1. In Study 2, over 75% of participants responded that neither being female nor being in a minority race should count in favour of or against a patient. A substantial percentage of participants in Study 1 thought economic and social statuses should not matter. In Study 2, these features took the form of patients who are celebrities and politicians, and over 75% participants responded that these features should not be used in triage.

The results of Study 2 also suggested that the mental availability of a feature is not the same as its strength. One observation in support of this view is that pregnancy, which was found by 86% of participants in Study 2 to be a favourable feature to the patient, was not even in the top 10 features of relevant features in Study 1. Another crucial question raised by the results in Study 1 was whether or not age, the top morally relevant feature, was a proxy for other medical features or was seen as relevant independently of other medical features. Study 2 at least partly answered this question. In Study 2, while age was found to be a relevant feature, its mean, while statistically shown to be non-zero, was only 0.47, so the responses were not as conclusive as directly medical features such as dying soon without a ventilator (M = 2.02), severity of symptoms (M = 1.62), and life expectancy (M = 1.30).

## General discussion

This study examined the views of the UK public on which and how strongly patient features should be considered in ventilator triage due to COVID-19 (and how strongly they should factor in favour or against patients). As a reference point, we compare the views expressed by our participants to the guidance issued by the British Medical Association. The BMA guidance was first published in April 2020, just a month after the COVID-19 outbreak was recognized by the World Health Organization as a pandemic [14]. The BMA guidance was specifically chosen because it was one of the few guidelines that explicitly addresses triage from an ethical perspective [12]. It was also discussed from the legal perspective [13]. The guidance states that when resources are severely constrained, admission to intensive care should be determined by the maximisation of medical benefits, and who should receive intensive care should be largely a function of the factors that will influence the patient’s probability of surviving and recovering from the intensive treatment.

Before we discuss how our data line up with the guideline, we must keep in mind how the timing of the surveys could have affected the opinions of our participants. Our second survey was conducted in November and early December, just between the first and second big wave in the UK. According to the data provided by the British government [11], the United Kingdom was experiencing a downward trend in COVID-19 cases. At this point, much of the anxiety about overwhelmed health systems had somewhat abated, and our participants might have believed that there was ample ICU capacity. The BMA guidance, on the other hand, was published to address direr situations in which medical resources are severely constrained. Even though we asked our participants to answer the survey under the hypothetical scenarios in which there is not enough ICU beds, it is possible that the then current state of affair in the world had implicitly influenced their answers.

With that acknowledged, our results suggest that the views of our participants support, but also diverge from some of the guidelines set by professional communities, such as the British Medical Association. As mentioned earlier, maximising medical benefits to the patient is the guiding principle that underlies the triage guidance set by British Medical Association, and we saw a similar concern expressed by our participants in our surveys, particularly the first open-ended survey. However, in the second survey, the factors rated most strongly in favour of patient selection do not necessarily coincide with BMA guidance. Respondents cited urgency of needing treatment, extreme discomfort due to severity of symptoms, and longer life expectancy. Urgency of needing treatment might coincide with maximizing benefit, since giving a ventilator to a patient who could comfortably wait might lead to preventable death. However, those with the highest medical need and most severe symptoms may also have a very low chance of survival. Longer life expectancy could potentially be justified within the utilitarian framework, assuming a notion of utility that includes years of life gained, but this might penalize older patients.

It is interesting that in the second survey there was relatively low support for the idea that a patient’s low chance of survival should count against allocation, with only 51% responding that this should count against the patient, and 28% responding that it should count in favour, with 21% neutral. One interpretation is that this finding is a result of a clash between different moral intuitions. The disapproving participants could be motivated by the utilitarian intuition that giving the ventilator to a patient with a low chance of survival will lead to lower medical benefits. The approving participants might have interpreted the low chance of survival as reflecting someone who is worse off and therefore should be prioritised.

Age and frailty might seem related to who is worse off or to their value of survival. The British Medical Association, along with other triage guidance documents, advised against penalizing older patients without considering comorbidities and prognosis [2]. Our participants seemed to agree, as only 21% thought being older should count against a patient, compared to 33% when a patient was very frail. This is consistent with a previous finding, in which UK survey participants showed a preference for an older but less frail patient than one who is younger but more fail [4]. In the present study, a considerable percentage (43%) of participants thought that frailty should count in favour of the patient being allocated the ventilator, but these participants might still prefer a less frail patient when forced to choose between two equally old patients but with different degrees of frailty.

Some factors concern effects on others, including foetuses. Even though pregnancy was not explicitly mentioned by the British Medical Association guideline, in view of the finding that COVID-19 infection substantially raised the risks of complication and mortality in pregnant women [9], it is likely that the public perception of pregnancy as morally relevant and positive is in line with expert opinions.

One feature that clearly deviated from the guideline but was found to be morally relevant and favourable by our participants was having several dependents. In Study 2, we asked the participants whether having young children or elderly family members in need of care should count in favour or against of the patient. 57% of participants thought this feature should count in favour of a patient, while 41% were neutral. In Study 1, 35.7% of participants also suggested that having dependents is a morally relevant feature. Some participants might have based this answer on the straightforward utilitarian calculation that the loss of the provider would cause additional losses of utility for their dependents. A compatible interpretation of these results is that our participants perceived childcare and eldercare as essential services. The BMA did acknowledge that once the distribution of resources could no longer be decided based on medical utility, priority should be given to workers in essential services so that social disruption is limited [7]. However, BMA limited essential services to physical utilities such as transportation, electricity, and water. Considering our results, an argument could be made that the notion of essential service ought to include childcare and eldercare, and that those who are responsibility for providing childcare and eldercare ought to receive priority. The persuasiveness of such an argument depends, however, on the extent to which one thinks the public’s views ought to influence policy making.

The British Medical Association appealed to the social good to justify prioritizing essential workers, which includes all hospital workers, regardless of whether they work with COVID-19 patients. This intuition was not shared by as many participants as might have been expected. Only 44% responded positively when the patient is a healthcare professional but does not work with COVID patients, with 53% being neutral. In contrast, 69% of participants appeared to be in favour of prioritizing those who have an active role in helping others with COVID-19. The preference for these workers could be motivated by a utilitarian intuition to maximize expected utility (by lowering the probability of infection of people associated with high degrees of medical utility) or a sense of reciprocity toward workers who have engaged in COVID-related care.

Our findings seem to have interesting implications regarding bias against disabled people, who face unique barriers in seeking medical help during the pandemic [15]. In a previous discrete choice experiment, people had shown small but statistically significant preference to people who are not physically or mentally disabled when deciding on how ventilators should be allocated [16]. In contrast, we did not see a clear indication of a bias against disabled patients in our studies. For example, 43% of survey 2 participants thought that being disabled should count neither in favour or against a patient, followed by those who thought it should count in favour (37%), and finally those who thought it should count against (20%). In our study, then, only a small minority thought disabled patients ought to be deprioritised. Nevertheless, our finding is consistent with the possibility that our participants who responded neutrally in our survey would have decided against disabled patients if they had to choose between a disabled patient and one that was not, so our findings did not completely rule out such bias.

While many of our findings conform to traditional utilitarian principles, they are also consistent with participants following certain non-utilitarian ethical principles. For instance, consider the “fair innings” argument [17, 18] which states that a member of a society is entitled to a certain length of life years, so anyone who has yet to receive this entitlement should be prioritised. The implication is that younger people in general should receive priority over older people when it comes to resource allocation. Participants in study 2 could have been thinking in terms of “fair innings” when they answered that being older should count against a patient (21%). Another non-utilitarian position is the severity approach [19], which states that, other things being equal, priority should be given to those with more severe medical conditions, because they are medically worse off. This approach is consistent with the behaviours of participants who prioritised, for instance, patients who are dying soon (83%) and those who were experiencing extreme discomfort (77%). Some responses were also consistent with the desert-based approach, which prioritise distribution based on their contribution to society [20]. Some participants favoured patients who had made more past contributions (28%) or have more potential to make future contributions (31%).

Another factor that might be related to desert is the circumstances under which the patient contracted the virus. For instance, a considerable number (40%) of participants thought that being responsible for contracting the virus should count against a patient, and 44% expressed similar attitude toward engaging in needlessly risky behaviours. The current access to vaccines, which was not available when data was collected, might increase these numbers further. These responses are especially pertinent in view of the recent phenomenon of hospitals running out of ICU beds due to unvaccinated patients [10]. Our participants’ negative reaction to certain features deviates substantially not only from the official guidelines but also potentially suggest a divergence from a wider utilitarian perspective, if participants believed that patients who willingly and unnecessarily engaged in risky behaviours should be deprioritised solely by virtue of this past behaviour. However, a utilitarian reading could construe those who responded negatively as treating past risky behaviours as a predictor for risk of future problems. If so, these participants were motivated by the lower expected utility of treating these patients.

On the other hand, participants’ attitude toward patients with a history of violent crime also cannot be straightforwardly reconciled with a general utilitarian perspective, since having committed violent crimes in the past does not directly influence the medical utility generated from treating the patient, such as chance of survival and life expectancy. 46% of our participants thought having a history of violent crimes should count against a patient. The numbers of negative responses were higher than either positive or neutral ones (though not together). Also of interest is the gap between the negative responses received for violent crimes and non-violent crimes (46% vs 21%). This gap cannot be explained by medical benefits, at least not in terms of direct medical benefits derived from treating these patients.

The divergence between the public view and the guideline raises an important question: What, if anything, do these popular opinions tell us about who should get a ventilator? We do not suggest that we should draw normative inferences directly from these surveys. The fact that many people found certain features to be morally relevant and positive does not prove that it is ethical to use that feature in allocation. Nevertheless, our findings become normatively and practically relevant on the assumption that hospital policies should conform somewhat to the moral standards of the public. Such an assumption could be argued on the ground that publicly funded hospitals, for instance, should be responsive to the values of the community. Separately, a degree of conformity between expert guidelines and moral views of the public is needed in order to maintain trust in the medical community. For such reasons, public opinion may be considered in designing and assessing public policies.

### Limitations

While Study 1 reduced framing bias by having participants spontaneously determine features that they believed would be relevant to allocation, this method could potentially miss relevant features that did not spontaneously come to participants’ minds, but that they nonetheless think are important. Study 2 found evidence of such omissions when features, such as pregnancy, were perceived as relevant and favourable, although they had been mentioned relatively infrequently in Study 1. Future studies should try to include more potentially relevant features that participants might overlook unless prompted.

Second, our study did not incorporate certain features that might be seen as relevant, including the hypothetical patient’s vaccination status (at the time of the initial survey, vaccines were not yet available). As vaccines become increasingly available and vaccination hesitancy becomes a core issue, vaccine status should be included in future studies.

Third, we only recruited opinions from a lay population. A potential future study could directly compare medical experts and the lay public in a two-sample study.

Fourth, while we suggested various underlying moral intuitions as hypotheses to explain the behaviours of the participants, these are not and cannot be substantiated by the current results, as the survey were designed to probe their views on resource allocation, and not the underlying motivations or justifications.

Fifth, our study did not involve a deliberative process, with discussion and information provided about the arguments for and against treating a feature as relevant. If such a process were presented to participants beforehand, they would potentially give different responses.

### Conclusions

Survey respondents perceived patients more favourably in hypothetical ventilator triage situations when the patients will die soon, are pregnant, have waited for a long time, are currently experiencing extreme discomfort, are expected to live longer, are COVID health workers, have dependents, wish to be put on ventilator, and are already on the only available ventilator. However, a considerable portion of the participants was also willing to potentially go beyond general utilitarian concerns by prioritizing ventilator allocation according to circumstances in which the patient contracted the virus, such as when the patient contracted the virus by needlessly engaging in activities with a high risk of COVID-19, and whether or not they have committed crime in the past. These results can be interpreted as illustrating that members of the UK public appear to broadly support the existing UK triage guideline in its focus on medical features and imperative to avoid discrimination based non-medical features, but also thought additional factors should be taken into account. It will be important for ethicists and policy makers to evaluate whether and how these additional features should be addressed in future iterations of triage policy.

## Supplementary Information

**Additional file 1. Additional survey texts, questions, and results**

## Data Availability

The datasets during and/or analysed during the current study are available from the corresponding author on reasonable request.
