# Supplementary material for: Which features of patients are morally relevant in ventilator triage? A survey of the UK public
Source: BMC Med Ethics. 2022 Mar 25;23:33. doi: 10.1186/s12910-022-00773-0 (PMC8956145; doi:10.1186/s12910-022-00773-0)
Supplement: Supplementary file 1 — Additional file 1. Additional survey texts, questions, and results [file 12910_2022_773_MOESM1_ESM.docx]

**Supplementary Material for “Which Features of Patients are Morally Relevant in Ventilator Triage? A survey of the UK public”**

Study 1

Introduction

People who develop severe breathing problems as a result of COVID-19 (or coronavirus disease 2019) will often die unless they are put on a mechanical ventilator. Ideally, there would be enough ventilators for all patients who need them. Unfortunately, there may not be enough ventilators available when there is a surge in the number of cases. In such circumstances, doctors sometimes must decide which of two patients in need should get the one available ventilator.

Question 1

In your opinion, which features of the patients in need morally **should** be considered when deciding who gets the ventilator if only one ventilator is available? This question is about what matters from a moral point of view. It is not asking about what is legal or about what is in your own self-interest. To say that a feature of patients morally **should** be taken into account means that it would be morally wrong for these features not to be taken into account or for them not to affect who gets the ventilator.

As an example, some people believe that the ventilator morally **should** go to the younger patient when other considerations are equal. These people could list “age” as one of their five features in their answer to this question. Other people believe that age morally should not have any impact on who gets the ventilator. These people should not list “age” as one of their five features in their answer to this question.

Please list five features that morally should be considered when deciding who gets the ventilator.

Why do you think that [a feature inputted by the participant] morally should be considered when deciding who gets the ventilator? (If you did not list a feature here, please explain why not.)

Question 2

In your opinion, which features of the patients in need morally **should not be considered** in deciding who gets the ventilator if only one ventilator is available? As before, this question is about what matters from a moral point of view. It is not asking about what is legal or about what is in your own self-interest. To say that a feature of patients morally **should not be taken into account** means that it would be **morally wrong for these features to affect who gets the ventilator** to be taken into account when deciding which patient should get the ventilator.

As an example, some people might believe the ventilator should always go to the younger patient when other considerations are equal. These people should not list “age” as one of their five features in their answer to this question. Other people might believe that age **should not have an impact** on who gets the ventilator. These people could list “age” as one of their five features in their answer to this question.  

**We are NOT asking for features that would indicate that someone should not get a ventilator. Instead, we are asking for features that should not have any effect one way or the other on whether a patient gets a ventilator.**

Please list five features that morally **should not be considered** when deciding who gets the ventilator.

Why do you think that [a feature inputted by the participant] morally should not be considered when deciding who gets the ventilator? (If you did not list a feature here, please explain why not.)

Study 2

Introduction

People who develop severe breathing problems as a result of COVID-19 (or coronavirus disease 2019) will often die unless they are put on a mechanical ventilator. Ideally, there would be enough ventilators for all patients who need them. Unfortunately, there might not be enough ventilators available when there is a large surge in the number of cases. In such circumstances, doctors sometimes must decide which patients in need will receive a ventilator and which patients will not receive a ventilator.

For the following question, we would like you to think about which features of patients a doctor should consider when deciding which patient will get a ventilator if only one ventilator is available and more than one patient needs a ventilator. The question is not about what is legal or in line with current policy but instead about what you believe morally should matter to this decision. Assume that patients are located in the UK, the treatment will be provided by the NHS, and all factors are equal other than the one that the question asks about.

The questions will be about how important a feature of a patient under consideration is and also the direction in which that feature should count. The options will always be:

This feature should count strongly in favour of the patient getting the ventilator.

This feature should count moderately in favour of the patient getting the ventilator.

This feature should count slightly in favour of the patient getting the ventilator.

This feature should not count at all either in favour of or against the patient getting

the ventilator.

This feature should count slightly against the patient getting the ventilator.

This feature should count moderately against the patient getting the ventilator.

This feature should count strongly against the patient getting the ventilator.

Although many answers will be controversial and you might be uncertain, we are asking for your personal opinion about what morally should matter to these difficult decisions; however, there will be no consequences or penalties for your responses, so you should respond freely based on your personal opinions and experiences.

Questions

These questions were presented in random order. The labels were not shown to participants.

Age: Some patients are younger, and others are older. Please indicate how important this feature is: the patient under consideration is older than the other patient who needs a ventilator.

Gender: Some patients are male, and others are female. Please indicate how important this feature is: the patient under consideration is female.

Race: Patients are from different races or different mixtures of race. Please indicate how important this feature is: the patient under consideration is in a minority race.

Disability: Some patients are moderately or severely disabled (for example, moderate or severe dementia), and others have no disability. Please indicate how important this feature is: the patient under consideration is moderately or severely disabled.

Low chance of survival: Some patients have a lower chance of surviving even if they are treated with a ventilator, while others have a higher chance of surviving if they are treated with a ventilator. Please indicate how important this feature is: the patient under consideration has a lower chance of surviving even if treated with a ventilator.

Dependents: some patients have young children, some have elderly family members who depend on them for care, while others have no dependents at all. Please indicate how important this feature is: the patient under consideration has several dependents.

Dying soon: some patients with COVID-19 will deteriorate and die very soon unless they are treated with a ventilator, while others may deteriorate and die later if they are not treated with a ventilator now. Please indicate how important this feature is: this patient will die very soon without a ventilator.

Extreme discomfort: some patients with COVID-19 have great difficulty breathing and are in extreme discomfort, whereas other patients have less severe symptoms now (though they are still at an equally high risk of dying without the ventilator). Please indicate how important this feature is: the patient under consideration is in extreme discomfort due to severe COVID-19 symptoms.

Patient wishes: some patients prefer not to be put on a ventilator, others prefer to be put on a ventilator, while some are unsure or have not expressed any preference about being put on a ventilator. Please indicate how important this feature is: the patient under consideration has expressed a preference to be put on a ventilator.

Past contributions to society: some patients have made more valuable contributions to society than others in the past. Please indicate how important this feature is: the patient under consideration has made valuable contributions to society in the past.

Future contributions to society: some patients are more likely to make future valuable contributions to society than others. Please indicate how important this feature is: the patient under consideration is likely to make valuable contributions to society in the future.

Obese: some patients are obese, some are underweight, while some have normal weight. Please indicate how important this feature is: the patient under consideration is obese.

Low quality of Life: some patients will have poorer quality of life even after successful treatment while some are expected to have higher quality of life after successful treatment. Please indicate how important this feature is: the patient under consideration is expected to have poor quality of life even after successful treatment.

Unnecessary risk: some patients contracted COVID-19 because they knowingly took part in unnecessary activities that increased their own risk of contracting COVID-19, while others avoided all needless risk but got COVID-19 anyway. Please indicate how important this feature is: this patient contracted COVID-19 from needless risky activities.

Infection responsibility: some patients are themselves responsible for catching COVID-19, so their medical condition is their own fault, whereas others are not at fault at all for catching COVID-19. Please indicate how important this feature is: the patient under consideration is responsible for catching COVID-19.

COVID-19 healthcare worker: Some patients are healthcare professionals who work with COVID-19 patients, some patients are healthcare professionals but do not work with COVID-19 patients, and some are not healthcare professionals at all. Please indicate how important this feature is: the patient under consideration is a healthcare professional who contracted COVID-19 from working with COVID-19 patients.

Non-COVID-19 healthcare worker: Some patients are healthcare professionals who work with COVID-19 patients, some patients are healthcare professionals but do not work with COVID-19 patients, and some are not healthcare professionals at all. Please indicate how important this feature is: the patient under consideration is a healthcare professional but does not work with COVID-19 patients.

Celebrity: some patients are famous celebrities, and others are not. Please indicate how important this feature is: the patient under consideration is a famous celebrity.

Politician: some patients are politicians, and others are not. Please indicate how important this feature is: the patient under consideration is a politician.

Waited for a long time: some patients have already waited for a ventilator for a longer period of time than others. Please indicate how important this feature is: the patient under consideration has already waited for a long time for a ventilator.

Physically fit: some patients are physically fit, and others are not. Please indicate how important this feature is: the patient under consideration is physically fit.

Non-violent crime: some patients have committed serious but non-violent crimes (such as car theft or credit card fraud) in the past, and some have not committed any non-violent crime. Please indicate how important this feature is: this patient committed a serious but non-violent crime in the past.

Violent crime: some patients have committed violent crimes (such as shooting or stabbing a victim) in the past, and others have not committed any violent crime. Please indicate how important this feature is: this patient committed a violent crime in the past.

UK Citizenship: some patients are citizens of the United Kingdom, and others are not citizens. Please indicate how important this feature is: the patient under consideration is a UK citizen.

Pregnancy: some patients are pregnant, while others are not. Please indicate how important this feature is: the patient under consideration is pregnant.

Mental health: Some patients have moderate or severe mental health problems (such as schizophrenia), and others do not. Please indicate how important this feature is: this patient suffers from moderate or severe mental health problems.

Long ventilator time: if treatment is started, some patients are expected to remain on a ventilator for weeks or months, whereas other patients are expected to need only a short time on a ventilator. A longer time on a ventilator potentially means that the ventilator will not be available to treat other patients. Please indicate how important this feature is: the patient under consideration is expected to need to stay on the ventilator for a particularly long time.

Longer life expectancy: some patients are expected to live for a longer period of time (e.g. many years) after successful treatment, while some are expected to live for a shorter period of time even after successful treatment. Please indicate how important this feature is: the patient under consideration is expected to live for a long time after successful treatment.

Frail: some patients are moderately or severely frail (in the sense that they have age-related loss of physical and mental function that makes them more prone to serious health complications), while some are not frail at all. Please indicate how important this feature is: the patient under consideration is moderately or severely frail.

Already on ventilator: some patients are already on a ventilator, and some have not yet been placed on a ventilator. If the patient is already on the ventilator, it would need to be removed so that another patient could be treated. Please indicate how important this feature is: the patient under consideration is already on the only available ventilator.

Attention Check: Some patients have features that differ from other patients. Just for this question, please select “This feature should count moderately in favour of the patient under consideration getting the ventilator” to convey that you are still paying attention.

Result

Table 3 contains the raw counts of responses (from “This feature should count strongly in favour of the patient” to “should count neither in favour or against” to “should count strongly against a patient”) for each feature.

| **Feature labels** | **3**  **Strongly in favour of a patient** | **2** | **1** | **0 - Neither** | **-1** | **-2** | **-3 Strongly against a patient** |
| --- | --- | --- | --- | --- | --- | --- | --- |
| Dying soon | 271 | 93 | 72 | 44 | 9 | 6 | 10 |
| Pregnant | 229 | 120 | 85 | 63 | 5 | 2 | 1 |
| Waited for a long time | 156 | 112 | 144 | 69 | 10 | 4 | 0 |
| Extreme discomfort | 165 | 119 | 108 | 97 | 10 | 6 | 0 |
| COVID health worker | 154 | 95 | 88 | 160 | 6 | 0 | 2 |
| Longer life expectancy | 112 | 111 | 123 | 148 | 4 | 4 | 3 |
| Dependents | 98 | 80 | 108 | 207 | 2 | 6 | 4 |
| Patient wishes | 84 | 93 | 101 | 207 | 10 | 7 | 3 |
| Already on ventilator | 125 | 64 | 73 | 169 | 43 | 17 | 14 |
| Non-COVID health worker | 68 | 77 | 80 | 270 | 6 | 2 | 2 |
| UK citizen | 90 | 68 | 51 | 277 | 7 | 6 | 6 |
| Physically fit | 63 | 70 | 75 | 233 | 51 | 9 | 4 |
| Future contribution | 50 | 59 | 50 | 335 | 4 | 0 | 7 |
| Age (older) | 65 | 52 | 78 | 204 | 84 | 12 | 10 |
| Past contribution | 39 | 47 | 55 | 349 | 4 | 3 | 8 |
| Disabled | 56 | 67 | 64 | 219 | 70 | 15 | 14 |
| Frail | 87 | 77 | 52 | 122 | 101 | 38 | 28 |
| Minority race | 39 | 41 | 28 | 283 | 8 | 0 | 6 |
| Long ventilator time | 70 | 51 | 29 | 210 | 82 | 29 | 14 |
| Mental health | 47 | 47 | 34 | 306 | 46 | 15 | 10 |
| Female | 29 | 31 | 17 | 416 | 3 | 3 | 6 |
| Politician | 26 | 31 | 15 | 395 | 11 | 5 | 22 |
| Obese | 40 | 43 | 42 | 254 | 81 | 28 | 17 |
| Celebrity | 20 | 26 | 13 | 414 | 11 | 2 | 19 |
| Low quality of life | 38 | 44 | 47 | 207 | 119 | 37 | 13 |
| Non-violent crime | 25 | 27 | 24 | 319 | 67 | 23 | 20 |
| Low chance of survival | 47 | 52 | 44 | 105 | 151 | 74 | 32 |
| Infection responsibility | 30 | 34 | 21 | 222 | 99 | 51 | 48 |
| Unnecessary risky activities | 29 | 31 | 30 | 193 | 114 | 51 | 57 |
| Violent crime | 24 | 25 | 23 | 202 | 76 | 66 | 89 |

Supplementary Table: Number of each response in each category, ordered response mean

Questionnaire

1. What is your current age? (18-99)
2. What is your gender?
   1. Male
   2. Female
   3. Other
   4. None/prefer not to answer
3. What is your ethnicity?
   1. Asian
   2. Black
   3. Hispanic/Latinx
   4. Native American
   5. Pacific Islander
   6. White
   7. Other
4. In which country do you currently reside? (Used for screening. Only participants who selected “United Kingdom” were allowed to proceed.)
5. Place yourself on one rung of this ladder to indicate your economic status (income and wealth), compared to others in your society. (0-100)
6. If the Conservative Party is typically understood as "right-wing" and the Labour Party is typically understood as "left-wing", where do you place yourself on the right-left wing scale below? (Extremely right-wing to extremely left-wing)
7. What is your self-identified religion?
   1. Christian/Catholic
   2. Christian/Non-Catholic
   3. Jewish
   4. Muslim
   5. Buddhist
   6. Atheist
   7. None
   8. Other
8. What is the highest degree or level of school you have completed? If currently enrolled, please mark the previous grade or highest degree received.
   1. Less than college or university
   2. College or university
   3. Post-graduate degree
9. How many of your parents are alive? (0,1,2, other, or unknown)
10. How many of your grandparents are alive? (0-4, other, or unknown)
11. How much do you know about COVID-19? (1-nothing to 7-as much as experts)
12. Are you a healthcare worker? (Yes/No/Unknown)
13. Are any of your close friends of family healthcare workers? (Yes/No/Unknown)
14. Have you had COVID-19? (Yes/No/Unknown)
15. Have any of your close friends or family had COVID-19? (Yes/No/Unknown)
16. Do you have any underlying health conditions that might make it worse than average for you to catch COVID-19? (Yes/No/Unknown)
17. Do any of your close friends or family have any underlying health conditions that might make it worse than average for you to catch COVID-19? (Yes/No/Unknown)
